# Application of Multivariate Statistical Techniques and Water Quality Index for the Assessment of Water Quality and Apportionment of Pollution Sources in the Yeongsan River, South Korea

**DOI:** 10.3390/ijerph18168268

**Published:** 2021-08-04

**Authors:** Md Mamun, Kwang-Guk An

**Affiliations:** Department of Bioscience and Biotechnology, Chungnam National University, Daejeon 34134, Korea; mamun1006001@gmail.com

**Keywords:** nutrients, organic matters, sewage treatment plants, summer monsoon, water pollution

## Abstract

This study assessed spatial and temporal variations of water quality to identify and quantify possible pollution sources affecting the Yeongsan River using multivariate statistical techniques (MSTs) and water quality index (WQI) values. A 15 year dataset of 11 water quality variables was used, covering 16 monitoring sites. The nutrient regime, organic matter, suspended solids, ionic contents, algal growth, and total coliform bacteria (TCB) were affected by the summer monsoon and the construction of weirs. Regression analysis showed that the algal growth was more highly regulated by total phosphorus (TP; R^2^ = 0.37) than total nitrogen (TN, R^2^ = 0.25) and TN/TP (R^2^ = 0.01) ratios in the river after weir construction and indicated that the river is a P-limited system. After constructing the weirs, the mean TN/TP ratio in the river was about 40, meaning it is a P-limited system. Cluster analysis was used to classify the sampling sites into highly, moderately, and less polluted sites based on water quality features. Stepwise discriminant analysis showed that pH, dissolved oxygen (DO), TN, biological oxygen demand (BOD), chemical oxygen demand (COD), chlorophyll-a (CHL-a), and TCB are the spatially discriminating parameters, while pH, water temperature, DO, electrical conductivity, total suspended solids, and COD are the most significant for discriminating among the three seasons. The Pearson network analysis showed that nutrients flow with organic matter in the river, while CHL-a showed the highest correlation with COD (r = 0.85), followed by TP (r = 0.49) and TN (r = 0.49). Average WQI values ranged from 55 to 141, indicating poor to unsuitable water quality in the river. The Mann–Kendall test showed increasing trends in COD and CHL-a but decreasing trends for TP, TN, and BOD due to impoundment effects. The principal component analysis combined with factor analysis and positive matrix factorization (PMF) showed that two sewage treatment plants, agricultural activities, and livestock farming adversely impacted river water quality. The PMF model returned greater R^2^ values for BOD (0.92), COD (0.87), TP (0.93), TN (0.91), CHL-a (0.93), and TCB (0.83), indicating reliable apportionment results. Our results suggest that MSTs and WQI can be effectively used for the simple interpretation of large-scale datasets to determine pollution sources and their spatiotemporal variations. The outcomes of our study may aid policymakers in managing the Yeongsan River.

## 1. Introduction

Rivers have been the most significant freshwater resources for human life, with the majority of ancient civilizations developing within river valleys, such as the Nile in Egypt, the Indo in India, and the Yellow River in China [1,2]. River water has numerous applications across all sectors of the economy, including for agriculture, industry, transportation, aquaculture, public water supply, and recreational and religious activities [1,3,4]; however, rivers have also been used for washing and disposal purposes since ancient times. Massive loads of industrial waste, domestic sewage, and agricultural byproducts make their way into rivers, contributing to significant declines in water quality [5,6]. Due to their role in carrying municipal and industrial wastewater as well as runoff from agricultural land, rivers are the most vulnerable water bodies to pollution and affect all levels of the food chain [7,8,9]; therefore, significant attention should be paid to river conservation.

Surface water quality in a given area is affected by both natural processes and anthropogenic factors, such as increasing exploitation of water resources [10]. Municipal and industrial discharge are continuous sources of pollution, while surface runoff is a seasonal phenomenon strongly affected by the climate of the watershed [1,3]. Seasonal variations in precipitation, surface runoff, inflows, and outflows directly influence river discharge and pollutant concentrations in river water [11]. As rivers provide the leading inland water resources for domestic, industrial, and irrigation purposes, it is vital to have reliable information about river water quality. Despite the dramatic development of water quality monitoring programs over the past few decades, representative and reliable water quality assessment remains difficult; therefore, gathering reliable information on river water quality, assessing spatial and temporal changes, performing source apportionment of pollution, and determining and controlling water pollution in rivers are indispensable tasks [9,12,13,14].

Water quality datasets are typically multifaceted, meaning it is of the utmost importance to develop a new way to statistically approach and interpret data with preventive or management purposes. There has been growing interest in studying such complex datasets using WQI values and MSTs among researchers, limnologists, and water quality managers in recent years [8,14,15,16,17,18]. WQIs and MSTs have played significant roles in water resource management to evaluate the quality of surface waters [3,9,19,20]. WQIs help clarify the general quality of a water source and have been applied in surface water quality assessments worldwide over the past few decades [21,22]. The primary objective of developing a WQI is transmuting a complex set of water quality data into a usable and straightforward value from which a layperson can determine the status of a water source [16,17,23].

WQIs have some limitations when used alone [9]. The combination of a WQI and MSTs may be useful for determining the water quality of rivers. MSTs such as cluster analysis (CA), stepwise discriminant analysis (DA), principal component analysis (PCA), factor analysis (FA), co-occurrence network analysis, and Mann–Kendall trend analysis are widely used for evaluating and interpreting both temporal and spatial variations in large and complex water quality datasets, as well as for source apportionment of pollution [1,3,6,8,9,24,25,26,27,28]. Mann–Kendall trend analysis has been applied to observe long-term patterns of water quality parameters [29]. CA has been employed in river water quality datasets to group similar sampling sites (spatial variability), while DA supports the statistical classification of samples and helps to group samples with common properties [24]. Network analysis is commonly used to identify relationships among river water quality variables [13]. PCA/FA is a dimensionality reduction technique that provides useful information about the most significant factors, resulting in a straightforward representation of the data [8]. PCA/FA is most commonly used to define data structures and provide qualitative information about potential pollution sources [3,30]; however, PCA/FA alone cannot define the quantitative contributions of the identified pollution sources to each variable. Receptor-based models, such as the positive matrix factorization (PMF) technique, can be used for this purpose [31,32]. The PMF technique has been used mainly for pollution source identification and apportionment in studies of the atmospheric environment [32]. The application of this technique to the apportionment of pollution sources in aqueous environments has increased in recent years [8,13,14].

The Yeongsan River is one of the four major rivers of Korea, which functions as a source for drinking, irrigation, industrial water, tourism attractions, and aquatic organism habitats. Recently, it has been impacted by intense land use, rapid population growth, and ongoing development [33]. It flows through Gwangju Metropolitan City and Naju City. Sub-basins that contain cities adversely impact river water quality and ecosystems due to their high contaminant levels relative to other sub-basins [33]. Summer monsoon rain directly increases inflow and outflow, regulating the nutrient load, ion content, suspended solids, organic matter, water clarity, and algal growth of the system [34]; thus, the summer monsoon season is a pivotal component affecting the ecosystem’s functional relationships among water quality parameters. In addition, weirs have modified the river ecosystem functions and increased the water residence time [29]. Consequently, algal growth has increased and cyanobacterial blooms have been observed in the river basin [29]. Furthermore, substantial water quality changes have occurred in the watershed due to the construction of two major sewage treatment plants (STPs) that process domestic and industrial wastewater from Gwangju Metropolitan City [29,33].

For these reasons, a detailed water quality assessment of the Yeongsan River is needed. This study aims to elucidate the spatial and temporal variations of water quality factors and identify water pollution sources in the Yeongsan River in South Korea. We analyzed the effects of the summer monsoon and weir construction on river water quality. The objectives of this study were to explore the co-occurrence network of river water quality parameters, identify long-term trends in water quality, extract information on the similarities or dissimilarities among sampling sites, determine which water quality variables are responsible for spatial and temporal variations in river water quality, identify the latent factors that explain the database structure, evaluate the effects of potential pollution sources (natural and anthropogenic) on water quality parameters, and estimate the contributions of possible pollution sources to the values of selected parameters.

## 2. Materials and Methods

### 2.1. Study Area

**Figure 1:**
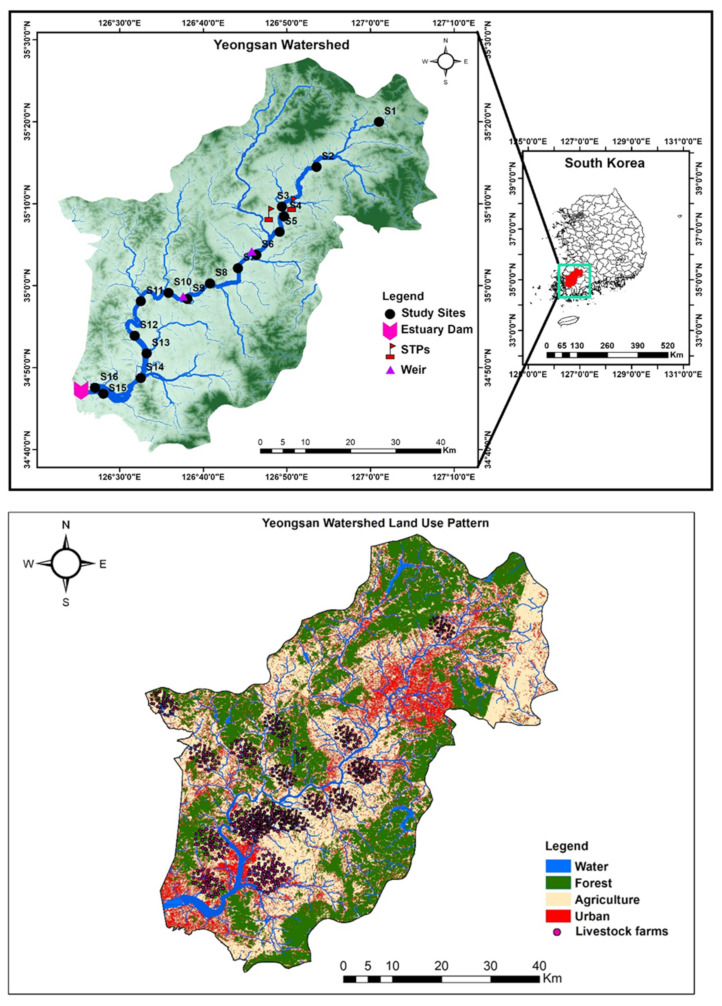
Maps showing water quality monitoring sites, land use patterns, livestock farms, and other facilities in the Yeongsan River basin in South Korea (STPs: sewage treatment plants).

The Yeongsan River is one of Korea’s four main rivers and lies in the southwest region of the Korean Peninsula [29], where it arises from the South Central Mountains (Figure 1). It flows into the southwestern sea after crossing agricultural areas and two megacities, Gwangju Metropolitan City and Naju City. The river has a length of 136.6 km and a watershed area of 3467.83 km^2^, consisting of 51% forest land, 34% agricultural land, and 7% urban areas [35]. Livestock farming is concentrated in the downstream portion of the watershed [29]. Two primary STPs process domestic and industrial wastewater from Gwangju City in the Yeongsan River basin [29]. Two large weirs were built in the river’s main stream in 2012, which broadened and deepened the river channel and increased the water residence time [24]. These three factors significantly affected water quality in the main stream of the Yeongsan River.

### 2.2. Data Sources and Analysis of Water Quality Parameters

Datasets from 16 water quality monitoring sites, comprised of 11 water parameters monitored monthly over 15 years (2005–2019; except for S5 and S10: 2007–2019, and S9: 2012–2019), were obtained from the Korean Ministry of Environment Water Information Network (http://water.nier.go.kr, accessed on 22 June 2021). The selected water quality parameters included hydrogen ion concentration (pH), water temperature (WT), dissolved oxygen (DO), electrical conductivity (EC), total suspended solids (TSS), total phosphorus (TP), total nitrogen (TN), biological oxygen demand (BOD), chemical oxygen demand (COD), chlorophyll a (CHL-a), and total coliform bacteria (TCB). A portable multi-parameter analyzer (YSI Sonde Model 6600) was used onsite to measure pH, DO, EC, and WT directly. The sampling, preservation, and analytical procedures for COD, BOD, TSS, TP, TN, CHL-a, and TCB were performed according to the national standards for South Korea [36].

### 2.3. Calculation of WQI

All water quality parameters (except WT) were used for the determination of the WQI. Calculation of the WQI was conducted through the following process, derived from the weighted arithmetic index method [23].

- Step 1: Calculate the unit weight factors for each parameter with the following formula:
  $$W_{n}=K/S_{n}$$
  where $K=1/\sum 1/S_{n}$ and *S_n_* = standard desirable value of the *nth* parameter. Upon summation of all selected parameters’ unit weight factors, *W_n_* = 1 (unity).
- Step 2: Calculate the sub-index (*Q_n_*) value using the following formula:
  $$Q_{n}=(V_{n}-V_{0})\;/\;\left(S_{n}-V_{0}\right)\times 100$$
  where *V_n_* = mean concentration of the *nth* parameter, *S_n_* = standard desirable value of the *nth* parameter, and *V_o_* = actual value of the parameter in pure water (*V_o_* = 0 for most parameters except for pH = 7 and DO = 14.6).
- Step 3: Combining steps 1 and 2, the *WQI* is calculated as follows:
  $$Overall\;WQI=\sum W_{n}Q_{n}/\sum W_{n}$$

The water quality status and possible water usages based on the WQI are shown in the Supplementary File (Table S1).

### 2.4. Statistical Analyses

**Figure 2:**
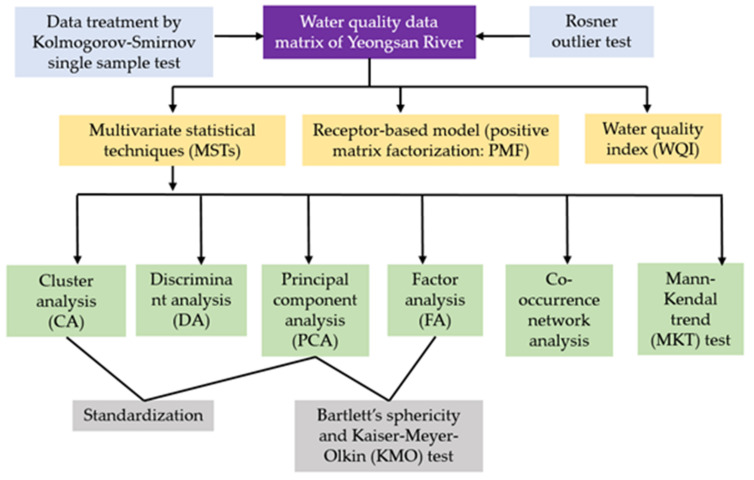
Methodological flow chart of the Yeongsan River data analysis.

The methodological approach to data analysis used in this study is presented in Figure 2.

#### 2.4.1. Co-Occurrence Network Analysis

To identify relationships among water quality parameters, a co-occurrence network analysis was conducted using Gephi software (Version 0.9.2). A Pearson-correlation-based network analysis was conducted with the water quality variables. To confirm the robustness of relationships among variables, we considered the Pearson correlation coefficient values significant at *p* < 0.05.

#### 2.4.2. Mann–Kendall Trend Analysis

The non-parametric Mann–Kendall test was first proposed by Mann [37] and improved by Kendall [38]. It was used to evaluate the temporal trends of water quality parameters in the Yeongsan River. This test has been widely applied to identify water quality trends in aquatic systems worldwide [29,39,40]. This analysis was performed using ProUCL version 5.1 software [41]. The results were reported as significant at a *p*-value threshold of 0.05 (significant difference, *p* < 0.05).

#### 2.4.3. Cluster Analysis (CA)

CA is a multivariate approach aimed primarily at grouping objects based on their features [6,24]. It categorizes objects using predetermined selection criteria so that each object is identical to all others in the cluster [15]. The resulting clusters of objects show high internal homogeneity (within the cluster) and high external heterogeneity (between clusters). One of the most common CA methods is Bray–Curtis similarity index clustering, which determines the level of similarity between any sample and the entire dataset and is usually illustrated as a dendrogram [42]. The dendrogram offers a visual depiction of the clustering processes and provides a simplified image of the groups and their proximity by dramatically reducing the dimensionality of the original data [6]. The spatial similarity and variability of water quality across the entire river basin were determined through CA. CA was performed using the PAST software [43].

#### 2.4.4. Discriminant Analysis (DA)

DA is used to classify cases into categorically dependent values, typically as a dichotomy [6]. If DA is useful for a given dataset, a classification table of correct and incorrect estimations will indicate a highly correct percentage [24]. Several quantitative attributes are used in DA to differentiate between two or more classes that occur naturally. In contrast to CA, DA allows for statistical classification of samples and is conducted with prior knowledge of the membership of objects in a specific group or cluster [30]. This method can be used after grouping samples that share similar properties, thereby obtaining discriminant functions for each group. In this study, three groups were selected for temporal (three seasons) analysis and three groups were selected for spatial (three sampling regions) evaluation, while a number of analytical parameters were used to impute monitoring results into groups (season or monitoring area). DA was performed on each raw data matrix using the standard and stepwise modes of constructing discriminant functions to assess the spatial and temporal variations of river water quality in the basin [6,9]. Grouping variables, including site (spatial) and season (temporal), were used as dependent variables, while the independent variables were the measured water quality parameters. DA analysis was performed using SPSS software (version 22.0; SPSS Inc., Chicago, IL, USA).

#### 2.4.5. Principal Component Analysis and Factor Analysis (PCA/FA)

PCA is a mathematical tool used to reduce the dimensionality of large datasets by building a covariance matrix of the original variables and extracting eigenvalues and eigenvectors (loadings or weightings) to obtain new orthogonal variables referred to as varifactors (VFs) through varimax rotation [1,8,24]. VFs are linear combinations of the original variables, and a given VF can include both potential and hypothetical variables. PCA is generally used to determine the minimum number of factors that account for the maximum variance in the dataset [6]. Finally, a few variables are identified that generally explain the vast majority of variance in the entire original dataset [24]. PCA/FA was used to obtain composite variables known as latent water pollution factors for the Yeongsan River in South Korea. PCA/FA analysis was performed using SPSS.

#### 2.4.6. Positive Matrix Factorization (PMF) Model

The PMF model is a multivariate factor analysis tool based on a least-squares approach that breaks down a sample data matrix into factor contributions and factor profile matrices [8,24]. It is an approach to factor analysis that specifically addresses the problem of non-optimal scaling. PMF is also one of the most important receptor models, and as such has been suggested for use in source apportionment by the US EPA and widely applied over the years in numerous research areas [8,13,24,32]. The following Equation (1) is used to describe the model:

(1) $$X_{ij}=\overset{p}{\underset{k=1}{\sum }}g_{ik}f_{kj}+e_{ij}$$

where *X_ij_* represents the elements of the input data matrix of *i* (number of samples) by *j* (chemical species) dimensions, *g_ik_* represents the elements of the factor scores, *f_kj_* represents the factor loading matrices, *e_ij_* is the residual for each sample or species, and *p* is the number of factors [24,44].

In summary, X=GF+e, where *X* is the data matrix based on sources and their contributions, *G* is the factor contribution, *F* is the factor profile, and *e* represents the residual matrix [13]. *G* and *F* are constrained to non-negative values due to the use of logarithmic penalty functions [45]. The object function *Q* must be minimized according to the PMF model [46], as shown in Equation (2):

(2) $$Q=\overset{n}{\underset{i=1}{\sum }}\;\overset{m}{\underset{j=1}{\sum }}\;[\frac{x_{ij}-\sum_{k=1}^{p}\;g_{ik\;}f_{kj}}{u_{ij}}]{\;}^{2}$$

where *u_ij_* is the uncertainty for the *jth* species in sample *i*. We used the following Equation (3) to determine the uncertainty of water quality parameters [47].

(3) $$Uncertainity=\sqrt[{\;}]{{\left(Error\;fraction\times Concentration\right)}^{2}+{\left(0.5\times MDL\right)}^{2}}$$

MDL is the method detection limit of the water quality parameter; the US EPA PMF-5 model was used for source apportionment of pollution in the Yeongsan River basin [31].

## 3. Results and Discussion

### 3.1. Physicochemical Properties of the River

**Figure 3:**
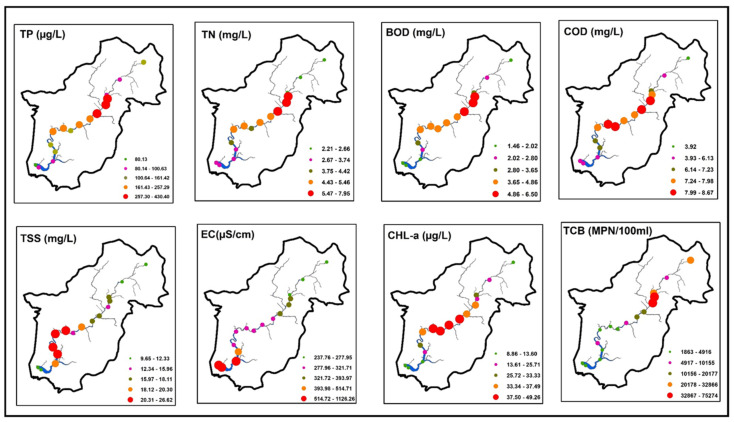
Spatial variations of water quality parameters (TP: total phosphorus; TN: total nitrogen; BOD: biological oxygen demand; COD: chemical oxygen demand; TSS: total suspended solids; EC: electrical conductivity; CHL-a: chlorophyll-a; TCB: total coliform bacteria). For site identification, please refer to Figure 1.

The average values of eight water quality parameters at 16 sampling sites along the Yeongsan River are shown in Figure 3. Nutrients (TP, TN) are the limiting factor for freshwater algal growth and are responsible for eutrophication [1,48]. The mean TP varied from 80.13 to 430.40 µg L^−1^ among sites S1–S16. Sites S5 (430.40 µg L^−1^), S4 (389.63 µg L^−1^), and S6 (371.53 µg L^−1^) showed the highest TP values, which were driven by wastewater discharge from two STPs for Gwangju City. Dodds et al. [49] proposed that TP concentrations > 75 µg L^−1^ indicate eutrophic rivers and streams. Mean TP levels above 75 µg L^−1^ were observed at all sites in this study. Mean TN values ranged from 2.21 to 7.95 mg L^−1^. As with TP, sites S5 (7.95 mg L^−1^), S4 (7.63 mg L^−1^), and S6 (7.01 µg L^−1^) showed the highest values of TN due to wastewater discharge from the two STPs. Dodds et al. [49] suggested that TN concentrations >1.5 mg L^−1^ indicate eutrophic rivers and streams. In the present study, mean TN concentrations were above 1.5 mg L^−1^ at all sites. As with TP and TN, the highest PO_4_-P (S4: 0.25 mg L^−1^, S5: 0.27 mg L^−1^, S6: 0.22 mg L^−1^), TDP (S4: 0.31 mg L^−1^, S5: 0.35 mg L^−1^, S6: 0.27 mg L^−1^), and NO_3_-N (S4: 3.10 mg L^−1^, S5: 3.0 mg L^−1^, S6: 3.01 mg L^−1^) were found in sites S4, S5, and S6 compared to other sites in the Yeongsan River due to wastewater discharge from the STPs (Table S2, Supplementary File).

High BOD (>5) and COD (>7) values indicate organic matter pollution associated with municipal, industrial, agricultural, and livestock wastewater effluents [1,50,51]. BOD values in the Yeongsan River ranged from 1.46 to 6.50 mg L^−1^. The highest mean BOD values were found at sites S4 (6.50 mg L^−1^), S5 (5.95 mg L^−1^), and S6 (5.61 mg L^−1^) due to wastewater discharge from the two STPs. Sites S15, S1, and S16 had BOD values < 2 mg L^−1^. The COD levels were >7 mg L^−1^ at sites S5 (8.67 mg L^−1^), S9 (8.52 mg L^−1^), S10 (8.36 mg L^−1^), S6 (8.28 mg L^−1^), S4 (7.98 mg L^−1^), S8 (7.80 mg L^−1^), S7 (7.79 mg L^−1^), and S11 (7.64 mg L^−1^) due to wastewater discharge and agricultural practices. The COD limit is 7 mg L^−1^ in water to be used for industrial purposes [51].

The movement of suspended solids in a river is part of the natural erosion and sediment transport processes [52]. The highest TSS value was observed at site S13 (26.62 mg L^−1^), where sandpits are near the river. Higher levels of dissolved ions in water cause higher EC. EC is a vital parameter because it directly affects the quality of water used for drinking and irrigation. Water with high EC can have an unpleasant mineral taste [53]; however, the potabilization process of the TSS and EC can amend the taste [53]. The average EC values in the Yeongsan River ranged from 237.76 to 1126.26 µS cm^−1^. The highest mean EC values were found at downstream sites, especially at S16 (1126.26 µS cm^−1^), S15 (996.4 µS cm^−1^), and S14 (890.53 µS cm^−1^), due to the reduced flow rate and estuarine environment. The threshold of EC > 300 µS cm^−1^ indicates severe pollution [51]. In the present study, mean EC levels were above 300 µS cm^−1^ at all sites (except S1, S2 and S3).

CHL-a is the central metric used to manage eutrophication in aquatic systems [54]. Mean CHL-a varied from 8.86 to 49.26 µg L^−1^. The highest CHL-a was found at site S10 (49.26 µg L^−1^). The United States Environmental Protection Agency [55] proposed that eutrophic rivers and streams are indicated by CHL-a concentrations >30 µg L^−1^. Mean CHL-a concentrations at nine sites (S10, S7, S9, S8, S11, S6, S5, S3, S12) were above 30 µg L^−1^. Microorganisms, particularly TCB, are commonly used as surface water pollution indicators due to their rapid responses to environmental changes [56]. TCB is generally associated with the coverage of commercial development in the watershed area. Sources of TCB include stormwater discharge, as well as agricultural and urban runoff. Surface water quality can be categorized into different pollution levels based on TCB values; TCB (most probable number per 100 mL, MPN mL^−100^) levels of ≤500, >500–10,000, >10,000–100,000, >100,000–1,000,000, and >1,000,000 indicate little pollution, moderate pollution, critical pollution, strong pollution, and excessive pollution, respectively [56,57]. The highest TCB levels were found at sites S5 (75,274.6 MPN mL^−100^) and S4 (58,871.9 MPN mL^−100^) due to wastewater discharge from the two STPs, indicating critical pollution levels. The remaining sites on the Yeongsan River showed moderate pollution levels based on TCB.

The results presented here show that domestic and industrial wastewater from two major STPs serving Gwangju City significantly impact water quality in the Yeongsan River. Moreover, the widespread distribution of agricultural lands and livestock farms throughout the watershed negatively affects the water quality of the river. Lee et al. [29] and Kang and An [58] noted that point source pollution from the two Gwangju City STPs and non-point source pollution from agricultural lands and livestock farms adversely impact the water quality of the Yeongsan River. The increased TP and TN concentrations of inflowing waters contribute to eutrophication and DO depletion [52]. High BOD and COD levels decrease DO levels in the water body, resulting in poor water quality and stress to resident aquatic life [48,59]. Higher TSS levels indicate a more contaminated water body from either natural or anthropogenic sources. TSS levels can affect fauna in a river, as egg-laying fish and invertebrates may not reproduce in areas with high sediment loads [52,60]. Eutrophic river conditions based on CHL-a can decrease the oxygen level and hinder ecosystem functions. If eutrophic conditions prevail in the river over a long period, macrophyte beds may completely cover the river surface [61]. Based on the observed abundance of TCB in the water, the microbiological quality of the water ranged from critical to moderate pollution levels. Based on our results, urgent measures should be taken to control water pollution in the Yeongsan River.

### 3.2. Monsoon Effects on Water Quality

Approximately half of the annual precipitation in Asia falls between July and August. This seasonality causes distinctive annual water quality patterns that are tightly coupled with longitudinal morphology [39]. In Korea, summer monsoon rain directly increases inflow and outflow while reducing water residence time and regulating nutrient loading to the aquatic system, water clarity, and algal growth [34,58,62]. The summer monsoon can be a critical factor affecting functional relationships among water quality variables in the ecosystem.

Kang and An [58] observed that rainfall intensity was higher during the monsoon season than any other time of the year. Previous research has demonstrated that monsoon rainfall has a negative impact on nutrient levels and a positive effect on TSS in the Yeongsan River [58,62]. This pattern indicates that rainfall can dilute nutrients and increase turbidity and that the high flushing rate of the monsoon season hinders algal blooms [62]. The present study showed that nutrients (TP and TN) and ionic dilution (EC) occurred in the river during the monsoon period (Figure S1, Supplementary File). During the monsoon period, nutrient co-limitation and algal washout were observed in the Yeongsan watershed. These results indicate that TSS and TCB concentrations increased during the monsoon period due to surface runoff from the watershed. The concentrations of algal chlorophyll are highly influenced by TP, TN, and TN/TP ratios depending on the seasonality in the Yeongsan River (Figure S2; Supplementary File). TP (R^2^ = 0.37) influenced the CHL-a level during the premonsoon season more than TN (R^2^ = 0.20) and TN/TP (R^2^ = 0.31) ratios. During the monsoon season, the same pattern was observed. In contrast, during the postmonsoon season, the CHL-a level was primarily influenced by TN (R^2^ = 0.45) compared to TP (R^2^ = 0.38) and TN/TP (R^2^ = 0.10) ratios. The empirical relationships between CHL-a-TP, CHL-a-TN, and CHL-a-TN/TP ratios based on the season indicated that TP is the better predictor for algal growth during premonsoon and monsoon seasons, while TN is the better predictor for primary productivity during the postmonsoon season. Additional studies are needed to fully elucidate the impacts of monsoons on the Yeongsan River’s water quality, and it is also crucial to reduce all other loads.

### 3.3. Effects of Weirs on Water Quality

The construction of weirs in streams and rivers can lead to longer water residence times and structural alterations of the physical environment, resulting in changes in chemical regimes, organic matter, ion contents, and algal growth [63,64]. Figure S3 (Supplementary File) shows the spatial effects of weir structures on the physicochemical quality of river water. TP concentrations decreased markedly (~2.74-fold) after weir construction in the Yeongsan River. This change was caused by phosphorus sedimentation from the water column due to structural changes from a lotic system to a lentic system, along with effects from river dredging and new phosphorus treatment facilities at the two STPs [29,65]. Like TP, TN concentrations decreased (~1.22-fold) after weir construction. The TN decrease appears to be closely associated with increased water residence time and new nutrient treatment facilities at the two STPs [29,65]. Despite significant reductions of TP and TN in the river, it remains under eutrophic conditions [49]. The present findings are in line with previous studies of the Yeongsan River [58,62]. Prior to weir construction, TN/TP observations indicated co-limitation by nitrogen and phosphorus in the river; however, after weir construction, phosphorus limitation of algal growth was observed (TN/TP ratio > 20). The CHL-a concentration increased after weir construction in the river due to increased water residence time and reduced washing out of the water column [65]. Increases in algal biomass and abundance have been observed in other freshwater systems impounded by dams or weirs [64,66]. These algal blooms can lead to water quality problems affecting agricultural, residential, and industrial water supplies [67]. We anticipate that the Yeongsan River’s impounded reaches have become more favorable to cyanobacteria than other algal groups after damming. Shifts toward cyanobacteria dominance associated with damming were observed in other river systems due to the higher resistance to xenobiotics of cyanobacteria [62,66]. The TSS concentration in the river also decreased (~1.37-fold) after weir construction due to settling in the impounded waterbody. In addition, EC decreased (~1.19-fold) in the river water due to dilution effects caused by increased water volume after weir construction. COD increased significantly (~1.18-fold) in the Yeongsan River, indicating that non-biodegradable organic matter became more abundant due to inputs of the various point source and non-point source pollutants [29]. Retention of organic matter in impounded waters has been observed worldwide in other freshwater systems [68]. The TCB level increased (1.29-fold) in the river. This was associated with the percentage of land containing commercial development in the watershed area, agricultural and urban runoff, livestock farms, and wastewater discharge [29]. Moreover, empirical model research based on nutrients (TP, TN) and CHL-a has been carried out to determine nutrient reduction targets in the aquatic systems [69]. The empirical regression analysis showed that TN/TP ratios were the better predictor for algal growth in the Yeongsan River for the periods before weir construction (Figure S4; Supplementary File). In contrast, after weir construction, the algal growth was highly influenced by TP (R^2^ = 0.37) than TN (R^2^ = 0.25) and TN/TP (R^2^ = 0.01) ratios in the river, indicating that the river is a P-limited system. The present findings confirm previous studies in impounded river ecosystems [63,65]. Mesocosm experiments and ecological modeling studies are needed to fully clarify the long-term effects of weirs at the ecosystem level.

### 3.4. Correlation Network of Water Quality Parameters 

**Figure 4:**
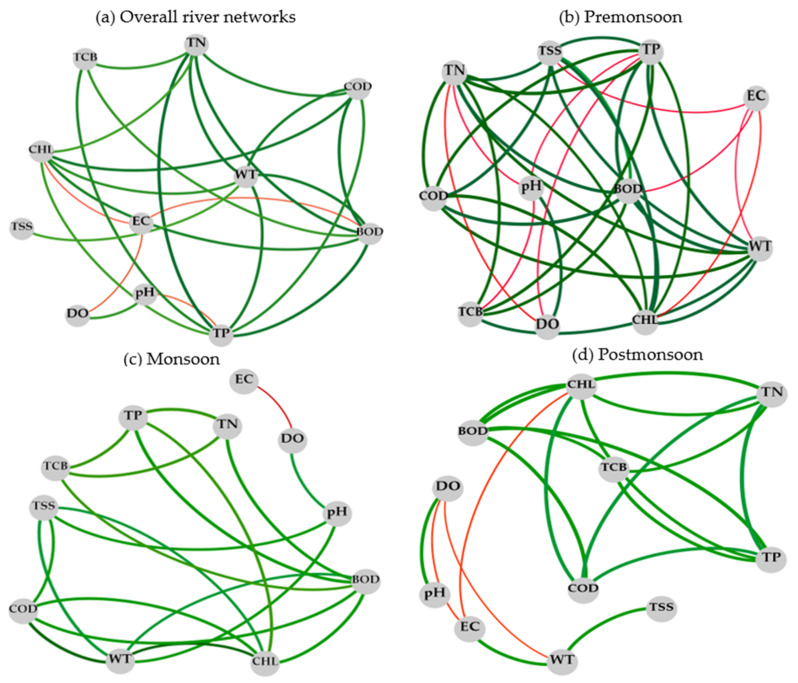
Co-occurrence correlation networks of water quality variables at spatial and temporal scales in the river (pH: hydrogen ion concentration; WT: water temperature; DO: dissolved oxygen; EC: electrical conductivity; TSS: total suspended solids; TP: total phosphorus; TN: total nitrogen; BOD: biological oxygen demand; COD: chemical oxygen demand; CHL-a: chlorophyll-a; TCB: total coliform bacteria; (**a**) overall river networks: from S1–S16; (**b**) premonsoon: January–June; (**c**) monsoon: July–August; (**d**) postmonsoon: September–December; red line indicates negative relation and green line indicates positive relation).

A Pearson correlation network was used to identify significant relationships among water quality parameters in the Yeongsan River (*p* < 0.05; Figure 4). The analysis of water quality parameters in the overall river network showed that pH is positively correlated with DO (r = 0.58), indicating high photosynthesis rates increase water pH (Figure 4a), in agreement with previous research [70]. A negative correlation was found between pH and TP. WT showed strong positive relationships with BOD, COD, TP, TN, and CHL-a. EC showed negative relationships with DO, BOD, and CHL-a. The BOD concentration had strong positive correlations with COD (r = 0.87), TN (r = 0.87), TP (r = 0.89), CHL-a (r = 0.75), and TCB (r = 0.56), while the COD concentration was positively related to TP (r = 0.67), TN (r = 0.74), and CHL-a (r = 0.85). CHL-a showed the strongest correlation with COD (r = 0.85), followed by TP (r = 0.49) and TN (r = 0.49). Similar results were obtained by Jung et al. [71], Song et al. [72], and Lee et al. [29], who observed that algal blooms feeding on high levels of inorganic nutrients increase organic matter levels in the river. TP, TN, and BOD have strong positive relationships with TCB, suggested that TP and TN have major positive effects on TCB growth. Previous studies have shown that nutrient and organic matter enrichment can stimulate TCB growth in the watershed, determining water usability [58,73]. Further, regression analysis among TCB–TP, TCB–TN, and TCB–BOD showed that TP, TN, and BOD explained 40%, 29%, and 26% of TCB variations in the Yeongsan River, respectively (Figure S5; Supplementary File). During the premonsoon season, TP (r = −0.55) and TN (r = −0.57) were negatively correlated with DO, indicating that nutrients were responsible for oxygen depletion (Figure 4b). Moreover, regression analysis of DO–TP and DO–TN during the premonsoon period showed that TP and TN explained 30% and 32% of DO variations in the river (Figure S6; Supplementary File). Our results showed that nutrients contribute to the development of hypoxia in the Yeongsan River. Previous research indicated that elevated TP and TN loads in streams and rivers could lead to severe hypoxia [50,74,75]. Notably, TSS was strongly related to CHL-a (r = 0.76) during the premonsoon season. During the monsoon period, EC had a negative relationship with DO (Figure 4c). WT was responsible for DO depletion (r = −0.75) in the Yeongsan River during the postmonsoon season (Figure 4d).

### 3.5. Water Quality Monitoring and Water Quality Index

The Mann–Kendall test was used to study long-term monitoring trends in water quality parameters throughout 2005–2019. The results of the trend analysis are presented in Table S3 (Supplementary File). BOD concentrations showed a decreasing trend, while COD exhibited an increasing trend in the river. The observed increase in the COD level appeared to be due to an increase in organic non-biodegradable substances driven by industrial growth and increasing population density, which led to continuous waste inputs into the river [29]. Previous studies have reported that high COD levels are caused by high levels of non-biodegradable organic wastewater, increased inputs of several non-point source pollutants such as humic substances during rainfall, and internal production by algae and aquatic plants [29,42,76]. As with the Yeongsan River, the concentrations of COD are increasing in the Nakong, Han, and Geum Rivers, while BOD is declining [12,42,77]. The CHL-a concentration in the river has been growing, and more frequent algal blooms have occurred in recent years. These blooms are due to increased water residence times caused by the construction of two weirs in the river’s main stream. TP showed a decreasing trend in the Yeongsan River over time due to new treatment facilities that began service at both STPs along the Yeongsan River’s main stream in October 2012 [78]. As with TP, the concentration of TN also showed a decreasing trend in the river over time. Sites S4 and S5 showed increasing trends in TCB due to wastewater discharge.

**Figure 5:**
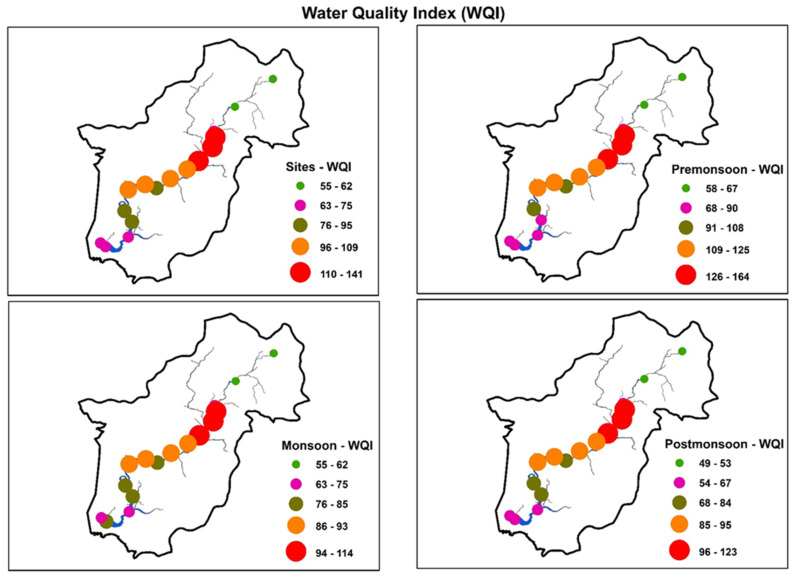
Water quality status at spatial and temporal scales in the river (premonsoon: January–June; monsoon: July–August; postmonsoon: September–December). For site identification, please refer to Figure 1.

The WQI enables general analysis of water quality at several levels that may affect the ability of a stream or river to host life to determine whether the overall quality of the waterbody poses a potential threat related to water usage [17,53]. The calculated WQI values for the Yeongsan River are presented in Figure 5. The results showed that the majority of the study sites along the Yeongsan River were in the very poor (WQI > 75–100) and unsuitable (WQI > 100) water quality categories. Average WQI values in the Yeongsan River ranged from 55 to 141. The highest WQI values were observed at sites S5 (141) and S4 (138). The unsuitability of sites S5 and S4 was primarily due to increased surface runoff from surrounding urban centers and direct discharge of wastewater from two STPs into the river. The mean WQI values at sites S1, S2, S3, S15, and S16 indicate poor water quality. The river’s water quality was very poor during the premonsoon season as compared to the monsoon and postmonsoon seasons. The WQI values for sites S1, S2, S3, S15, and S16 indicated that the water is only suitable for irrigation and industrial purposes.

High WQI values at the remaining sites in the river are driven mainly by various anthropogenic activities, including direct wastewater inflow from industrial and residential facilities, agricultural runoff, direct drainage of untreated water from small-scale industrial sites and factories, and persistent dumping of solid waste by communities living alongside the river [17,29]. Similar results were obtained in the Cauvery River and Himalayan rivers and streams due to the discharge of domestic sewage [79,80]. Seasonally, WQI values were higher in the premonsoon period than in the monsoon and postmonsoon seasons. This pattern indicates that the discharge of concentrated wastewater without dilution leads to high WQI values in the premonsoon season [70]. Similar seasonal variations of WQI were reported by Hemamalini et al. [81] in India and Ahmed et al. [22] in Iraq. Spatially, greater differences were observed in WQI at sites S4 and S5, which may have been driven by point source pollution effects from the two STPs. Sites S1 and S2 showed low WQI values, while S4 and S5 showed high WQI values due to direct discharge from the two STPs. Overall, WQI values were very high and may pose a risk based on the intended use of the water.

### 3.6. Spatial Similarity and Site Grouping

**Figure 6:**
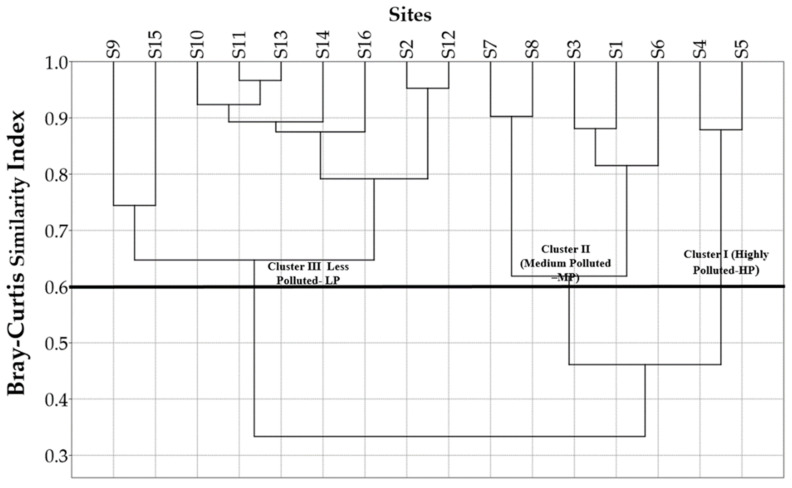
A dendogram showing clustering of sampling sites according to surface water quality characteristics of the Yeongsan River basin.

CA was used to construct similarity groups among the sampling sites. This method creates a dendrogram in which all 16 sampling sites along the river are grouped into three statistically significant clusters based on a 60% Bray–Curtis similarity index threshold (Figure 6). Cluster I comprised sites S4 and S5, which are the two highly polluted (HP) sites. These sites receive pollutants directly from the two wastewater disposal plants, along with urban runoff. Cluster II included sites S6, S1, S3, S8, and S7, which were moderately polluted (MP) sites. These sites are affected by agricultural and livestock activities. Cluster III comprised sites S12, S2, S16, S14, S13, S11, S10, S15, and S9, which are downstream sites (except site S2), which were less polluted (LP). This finding suggests that dilution and purification processes improve water quality with increasing distance from upstream pollution sources [29]. The present results show that the CA technique is useful for ensuring accurate surface water classification across the study region and allows for the development of an optimal spatial sampling strategy to reduce the number of sampling stations and associated expenses [6]. Other researchers have successfully utilized the CA approach to identify similarities among sampling sites and track water quality programs [1,3,6,29]. The CA approach can also be used for spatial DA [9].

### 3.7. Spatial and Temporal Variations in River Water Quality

Spatial DA was conducted on the raw dataset comprised of 11 water quality parameters after grouping sites into the three critical classes of HP, MP, and LP based on CA. The clustered sites were the dependent variable, and all measured water quality parameters were considered independent variables. The spatial discriminant functions (DFs) and classification matrices (CMs) used in this study are provided in Tables S4 and S5 (Supplementary File), respectively. The spatial standard mode DFs based on 11 discriminant parameters yielded corresponding CMs that assigned 80.8% of cases correctly, while the spatial stepwise DA mode provided CMs with 82.1% correct assignations with only seven discriminant parameters. Stepwise DA indicated that pH, DO, TN, BOD, COD, CHL-a, and TCB are important parameters for spatial discrimination.

**Figure 7:**
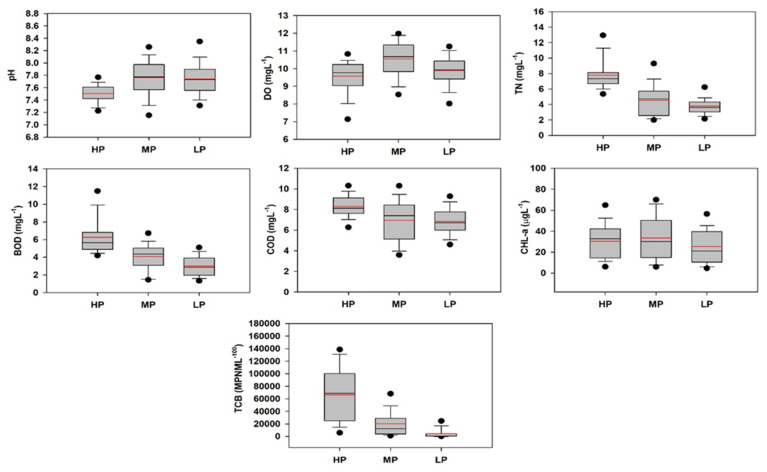
Spatial variations in pH, DO, TN, BOD, COD, CHL-a, and TCB in the Yeongsan River (pH: hydrogen ion concentration; DO: dissolved oxygen; TN: total nitrogen; COD: chemical oxygen demand; CHL-a: chlorophyll-a; TCB: total coliform bacteria; HP: highly polluted; MP: medium polluted; LP: less polluted).

To evaluate the patterns associated with spatial variations in river water quality, box and whisker plots of the discriminating parameters identified through spatial stepwise DA were created (Figure 7). The average pH was slightly lower in the HP region, with substantial spatial variations, suggesting that hydrolysis of acidic materials caused a decrease in pH [14]. The natural pH values >7 were due to the water contacting calcareous soil containing Ca^2+^ and HCO_3_^−^ in the area studied [14]. The HP sites had higher average BOD and COD values, indicating much more severe organic pollution. Such pollution results in the depletion of DO at the HP sites. In addition, greater average TN was found at HP sites, suggesting that eutrophication may be a severe water quality problem in the area. Average TCB levels were high at HP sites, suggesting a critical level of microbial contamination. In summary, the spatial relationships between variables showed that environmental pollution problems were worse at HP sites than at MP and LP sites; therefore, the HP sites should be the focus of remediation efforts.

Temporal DA was performed on the raw dataset after dividing the original dataset into three seasonal groups (premonsoon, monsoon, and postmonsoon). The temporal DFs and CMs are shown in Tables S6 and S7 (Supplementary File), respectively, and were used to evaluate seasonal changes in water quality in the Yeongsan River. Temporal standard DFs using 11 discriminating variables yielded corresponding CMs that assigned 81.3% of cases correctly; however, in stepwise temporal mode, DA returned CMs with 80% correct assignment using only six discriminating parameters. The temporal results indicated that pH, WT, DO, EC, TSS, and COD are the most significant parameters for discrimination among the three seasons. This finding suggests that most temporal variations of river water quality are associated with these six parameters.

**Figure 8:**
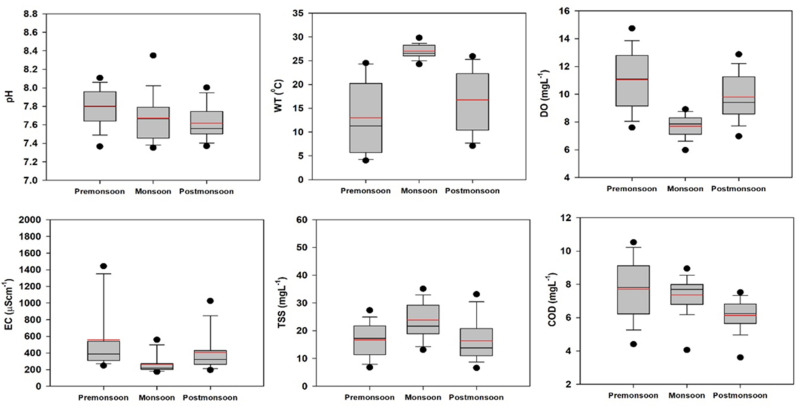
Temporal variations in pH, WT, DO, EC, TSS, and COD in the Yeongsan River (pH: hydrogen ion concentration; WT: water temperature; DO: dissolved oxygen; EC: electrical conductivity; TSS: total suspended solids; COD: chemical oxygen demand; premonsoon: January–June; monsoon: July–August; postmonsoon: September–December).

As determined through DA, box and whisker plots of selected parameters with seasonal trends are presented in Figure 8. The average WT was highest during the monsoon season. A robust inverse relationship was observed between WT and DO, which was due to the influence of seasonality. This inverse relationship is a natural occurrence, as warmer water is more easily saturated with oxygen and can hold less DO [6]. Average EC was reduced during the monsoon season due to the dilution effect. TSS concentrations were elevated in the monsoon season due to high water flow. Average COD values were higher during the premonsoon season due to the low volume of river flow. Previous studies have successfully applied the DA method to identify discrimination parameters for spatial and temporal variations [1,3,6,9,14]. Overall, the DA methodology significantly reduced the original dataset and helped to define the parameters responsible for spatial and temporal variations.

### 3.8. Identification of Potential Pollution Sources

PCA/FA is a dimensionality reduction technique that provides information about the most significant factors by simplifying data; therefore, various studies have utilized this method to explore the pollution sources affecting a water system [6,7,14,15]. To assess data suitability prior to PCA/FA, the Bartlett and KMO tests were conducted. The results showed that KMO = 0.55, while Bartlett’s test was significant (*p* = 0.000), indicating that the data were appropriate for PCA/FA and that a meaningful relationship between the variables was present. PCA/FA with a varimax rotation led to the identification of three varifactors (VFs), which explained 85.58% of the total variance (Table 1). The absolute loading values were used to classify factor loadings as strong (>0.70) or moderate (0.5–0.7). A scatter plot of eigenvalues using PCA/FA allows us to identify three groups of the study sites and helps to explain the pollution source identification of the Yeongsan Rivers (Figure S7; Supplementary File).

Group-I (highly polluted) explains the first VF (VF1), accounting for 43.27% of the total variance, which had strong positive loadings for WT, TSS, BOD, COD, TN, and CHL-a. VF1 also showed moderate positive loading for TP. This factor represents nutrient and organic matter sources [9,15]. COD and BOD are indicators of organic matter pollution [27,59]. The highest mean values of COD and BOD were found at sites S4 and S5, which receive wastewater discharge from two STPs. TP and TN are indicators of nutrient pollution [12]. As with BOD and COD, the highest mean TP and TN values were observed at sites S4 and S5, which receive wastewater discharge from the STPs serving Gwangju City. The highest mean WT was also observed at sites S4 and S5. This factor represents the effects of nutrients and temperature on CHL-a. Furthermore, a negative contribution of DO to this VF is expected, as DO is negatively correlated with WT, BOD, COD, CHL-a, TP, and TN [1]; hence, the major sources of COD, BOD, TN, and TP are wastewater discharge from the two Gwangju City STPs. These sites are strongly affected by industrial effluent and domestic sewage; therefore, VF1 can be interpreted as representing point source pollution discharge.

Group-II (medium polluted) represents the second VF, which accounted for 25.31% of the total variance and had strong positive loadings for TP and TCB and moderate positive loadings for TN and BOD. This factor represents nutrient, organic matter, and microbial pollution. BOD and TCB originate from livestock farms, while TN and TP arise from agricultural runoff into the river. The strong negative loading of pH for this VF indicates that pH may regulate BOD in the water, as it is one of the main conditions affecting redox reactions of organic matter [9,14]; therefore, this factor could be interpreted as non-point source pollution associated with agricultural and livestock farming activities.

Group-III explains (less polluted) the third VF, which accounted for 17.0% of the total variance and had strong positive loading for DO. This VF represents the river’s physicochemical condition and biological state. This factor can be considered to represent oxygen-consuming organic pollution [14]. The pollution sources identified here differed from those reported in other studies. For example, water quality in the Langat River (Malaysia) was primarily affected by saltwater intrusion, agricultural and industrial runoff, and geological weathering [82]. Rivulet flux, surface runoff, and tidal flow were the major pollution sources in Aerial Bay [83]. The water quality variables selected were the principal determinants of the source type; therefore, for accurate characterization of the pollution in a waterbody, the variables to be analyzed must be chosen appropriately [14].

### 3.9. Source Apportionment Using the PMF Model

After identifying possible pollution sources, the contribution of each source to water quality variables was apportioned using the PMF method. After running the PMF model, the number of source factors was fixed to three based on the outcome of the PCA/FA. The results of the source apportionment for the Yeongsan River are presented in Table 2. As shown in this table, R^2^ values between observed and predicted water quality parameters were used to assess the accuracy of PMF model predictions. All water quality parameters (except DO and TSS) showed good linear regression results. Greater R^2^ values were detected for BOD (0.92), COD (0.87), TP (0.93), TN (0.91), CHL-a (0.93), and TCB (0.83) than for other factors. High R^2^ values indicate that the source apportionment results are reliable [8]. In the Yeongsan River, most variables were influenced primarily by point source pollution from STPs (Factor 1), including nutrients (TP: 46.03%, TN: 49.12%), organic matter (BOD: 62.56%, COD: 58.04%), suspended solids (TSS: 60.70%), and algal chlorophyll (CHL-a: 74.58%). Factor 2 was primarily characterized by BOD (32.22%), TP (46.24%), TN (34.50%), and TCB (90.32%); thus, factor 2 likely represents agricultural and livestock farming sources. Factor 3 was mainly characterized by EC (64.85%), as well as represented ions from untreated domestic sewage, industrial effluent, and agricultural runoff. Overall, the outcomes of the PMF model were in good agreement with the results of PCA/FA. This consistency indicates that PCA/FA combined with PMF is a versatile tool for source identification and source apportionment [8,13]. Although the PMF approach is commonly used in air pollution research, few previous studies have applied PMF to apportion pollution sources affecting water resources [8].

### 3.10. Management Implications and Recommendations

In this study, the spatiotemporal variations of selected water quality parameters in the Yeongsan River were evaluated for assessment of water quality as well as for identification and quantification of possible pollution sources. The observed spatial variations suggest that STPs serving Gwangju City play a major role in determining river water quality and are important sources of pollution. Temporal variations were more distinct during the monsoon season due to the dilution of nutrients and ions and increased turbidity. The high WQI values in all seasons at most river sites indicated very poor river water quality, making the water unsuitable for drinking and industrial purposes. Henceforth, precautions should be taken immediately in communities that use this river water as a source of drinking or industrial water. It is also essential to evaluate which metals species are present or dominant. Based on nutrient concentrations, the river was in a eutrophic state. Organic pollution was prominent in the river. The PCA/FA and PMF indicated that the presence of the two STPs, along with agricultural activities and livestock farming, impact the river water quality. The present findings indicate that the main pollution source in the Yeongsan River, namely wastewater disposal from the two STPs serving Gwangju City, should be the focus of pollution control efforts. The CA results presented in this study can be used to formulate effective sampling strategies for future monitoring programs and may help to identify areas of the rivers that are critically affected by natural and anthropogenic pollution. Water quality managers and water monitoring programs frequently face budget, time, and laboratory constraints that limit sample analysis. CA results can be used in such situations to reduce the number of sampling sites without losing vital information. DA is useful for studying the spatial and temporal variations of water quality. Moreover, the present study will support further research efforts to clarify the point and non-point sources of pollution in this watershed, the impacts of the monsoon and weir construction on river water quality, and the development of MST and WQI methods that can be used to assess pollution sources and spatiotemporal variations in river water quality.

To protect water quality in the Yeongsan River, the following steps must be taken: minimization of industrial and domestic effluent disposal from the two STPs; implementation of advanced and effective treatment technologies for treating wastewater prior to discharge into the river; control fertilizer use, use low-impact fertilizer, and control livestock wastewater in the catchment area; enforcement of strict water quality regulation measures; regular monitoring of river water to monitor its quality deterioration; formulation and implementation of effective management strategies.

## 4. Conclusions

Various multivariate statistical techniques, including CA, DA, PCA/FA, a receptor modeling technique (PMF), and a WQI were used to evaluate spatial and temporal variations of surface water quality and identify potential pollution sources in the Yeongsan River basin. CA allowed the 16 sampling sites to be grouped into three clusters with similar water quality characteristics. Spatial stepwise DA showed that all variables except WT, EC, TSS, and TP exhibit significant spatial variations due to anthropogenic activities. Temporal stepwise DA revealed that pH, WT, DO, EC, TSS, and COD showed substantial seasonal differences due to the strong seasonality of WT and water flow. The high WQI values observed in the river indicate very poor water quality. The effects of the monsoon suggested that rainfall could dilute nutrients and ions, increase turbidity, and hinder algal growth in the river. The Mann–Kendall test showed increasing trends for COD and CHL-a and decreasing trends for TP, TN, and BOD due to impoundment of the Yeongsan River. Empirical models of nutrients and chlorophyll-a and nutrient ratios indicated potential P limitations on algal growth in the river after weir construction. Network analysis showed that nutrients flow with organic matter in the river. PCA/FA and PMF showed that the two STPs, agricultural activities, and livestock farming influence river water quality.

## Figures and Tables

**Table 1:** Varimax-rotated component matrix for water quality parameters (Kaiser–Meyer–Olkin (KMO) = 0.55; Bartlett’s test was significant (*p* = 0.000)).

Variables	Components		
	VF1	VF2	VF3
pH	0.05	−**0.76**	0.42
WT	**0.85**	0.27	0.06
DO	−0.18	−0.21	**0.91**
EC	−0.39	−0.17	−**0.77**
TSS	**0.73**	−0.48	−0.11
BOD	**0.83**	**0.50**	0.16
COD	**0.92**	0.15	−0.01
TP	**0.67**	**0.71**	−0.02
TN	**0.74**	**0.61**	−0.16
CHL-a	**0.82**	−0.03	0.39
TCB	0.19	**0.82**	0.13
Eigenvalues	4.76	2.78	1.87
% of Variance	43.27	25.31	17.00
Cumulative %	43.27	68.58	85.58

Extraction method: principal component analysis. Rotation method: varimax with Kaiser normalization. Bold values represent strong (>0.70) and moderate loadings (0.5–0.7), respectively. Note: pH: hydrogen ion concentration; WT: water temperature; DO: dissolved oxygen; EC: electrical conductivity; TSS: total suspended solids; BOD: biological oxygen demand; COD: chemical oxygen demand; TP: total phosphorus; TN: total nitrogen; CHL-a: chlorophyll-a; TCB: total coliform bacteria.

**Table 2:** Source apportioning contributions for water quality variables in the Yeongsan River using the positive matrix factorization model.

Variables	Profile Contribution (Conc.)			Profile Contribution (%)			R^2^
	Factor 1	Factor 2	Factor 3	Factor 1	Factor 2	Factor 3	
WT	8.41	2.29	5.87	50.77	13.80	35.43	0.55
DO	5.13	1.16	3.59	51.93	11.75	36.32	0.12
EC	149.17	0.06	275.33	35.14	0.01	64.85	0.69
TSS	10.58	2.48	4.37	60.70	14.23	25.06	0.38
BOD	2.33	1.20	0.19	62.56	32.22	5.22	0.92
COD	4.10	1.29	1.68	58.04	18.23	23.73	0.87
TP	87.50	87.90	14.69	46.03	46.24	7.73	0.93
TN	2.13	1.50	0.71	49.12	34.50	16.38	0.91
CHL	21.58	7.23	0.12	74.58	25.00	0.41	0.93
TCB	2.83	13239.00	1415.80	0.02	90.32	9.66	0.83

(WT: water temperature; DO: dissolved oxygen; EC: electrical conductivity; TSS: total suspended solids; BOD: biological oxygen demand; COD: chemical oxygen demand; TP: total phosphorus; TN: total nitrogen; CHL-a: chlorophyll-a; TCB: total coliform bacteria).

## Data Availability

The data may be available upon request to the corresponding author, subject to the funding agency’s approval.

## Supplementary Materials

The following are available online at https://www.mdpi.com/article/10.3390/ijerph18168268/s1: Figure S1. Seasonal impacts on nutrients (TP: total phosphorus; TN: total nitrogen), nutrient ratios (TN/TP ratios), algal chlorophyll (CHL-a: chlorophyll-a), suspended solids (TSS: total suspended solids), ionic concentrations (EC: electrical conductivity), organic matter (COD: chemical oxygen demand), and total coliform bacteria (TCB) in the Yeongsan River (PreM—premonsoon: January–June; Mon—monsoon: July–August; PostM—postmonsoon: September–December). Figure S2. Regression analysis of log-transformed algal chlorphyll (CHL-a: chlorphyll-a) with TP (total phosphorus), TN (total nitrogen), and TN/TP ratios during premonsoon (January–June), monsoon (July–August), and postmonsoon (September–December) seasons in the Yeongsan River. Figure S3. Changes in nutrients (TP: total phosphorus; TN: total nitrogen), nutrient ratios (TN/TP ratios), algal chlorophyll (CHL-a: chlorophyll-a), suspended solids (TSS: total suspended solids), ionic concentrations (EC: electrical conductivity), organic matter (COD: chemical oxygen demand), and total coliform bacteria (TCB) in the Yeongsan River in the periods before and after weir construction (BWC: before weir construction; AWC: after weir construction). Figure S4. Regression analysis of log-transformed algal chlorphyll (CHL-a: chlorphyll-a) with TP (total phosphorus), TN (total nitrogen), and TN/TP ratios in the Yeongsan River in the periods before and after weir construction. Figure S5. Regression analysis of TCB (total coliform bacteria) with TP (total phosphorus), TN (total nitrogen), and BOD (biological oxygen demand). Figure S6. Regression analysis of DO (dissolved oxygen) with TP (total phosphorus) and TN (total nitrogen). Figure S7. Scatter plot of the eigenvalue scores for the sites in the Yeongsan River based on PCA/FA. Table S1. WQI range, status, and possible usage of the water sample (Brown et al. 1972). Table S2. The concentrations of PO_4_-P, TDP, and NO_3_-N at 16 sites in the Yeongsan River. Table S3. Mann–Kendall trend test results for water quality variables at 16 monitoring sites and overall river network (WT: water temperature; EC: electrical conductivity; TP: total phosphorus; TN: total nitrogen; TSS: total suspended solids; BOD: biological oxygen demand; COD: chemical oxygen demand; CHL-a: chlorophyll-a; TCB: total coliform bacteria). Table S4. Classification functions for discriminant analysis of spatial variations in the water quality of the Yeongsan River (pH: hydrogen ion concentration; WT: water temperature; DO: dissolved oxygen; EC: electrical conductivity; TSS: total suspended solids; TP: total phosphorus; TN: total nitrogen; BOD: biological oxygen demand; COD: chemical oxygen demand; CHL-a: chlorophyll-a; TCB: total coliform bacteria; HP: highly polluted; MP: medium polluted; LP: less polluted) (Fisher’s linear discriminant functions). Table S5. Classification matrix for discriminant analysis of spatial variations in water quality of the Yeongsan River (HP: highly polluted; MP: medium polluted; LP: less polluted). Table S6. Classification functions for discriminant analysis of temporal variations in water quality of the Yeongsan River (pH: hydrogen ion concentration; WT: water temperature; DO: dissolved oxygen; EC: electrical conductivity; TSS: total suspended solids; TP: total phosphorus; TN: total nitrogen; BOD: biological oxygen demand; COD: chemical oxygen demand; CHL-a: chlorophyll-a; TCB: total coliform bacteria; premonsoon: January–June; monsoon: July–August; postmonsoon: September–December) (Fisher’s linear discriminant functions). Table S7. Classification matrix for discriminant analysis of temporal variations in water quality of the Yeongsan River (premonsoon: January–June; monsoon: July–August; postmonsoon: September–December).
