# Supplementary material for: Application of Multivariate Statistical Techniques and Water Quality Index for the Assessment of Water Quality and Apportionment of Pollution Sources in the Yeongsan River, South Korea
Source: Int J Environ Res Public Health. 2021 Aug 4;18(16):8268. doi: 10.3390/ijerph18168268 (PMC8392859; doi:10.3390/ijerph18168268)
Supplement: Supplementary file 1 [file ijerph-18-08268-s001.zip › ijerph-1288832-supplementary.pdf]

Supplementary File

Application of Multivariate Statistical Techniques and Water Quality Index for the Assessment of Water Quality and Apportionment of Pollution Sources in the Yeongsan River, South Korea

Md Mamun and Kwang-Guk An

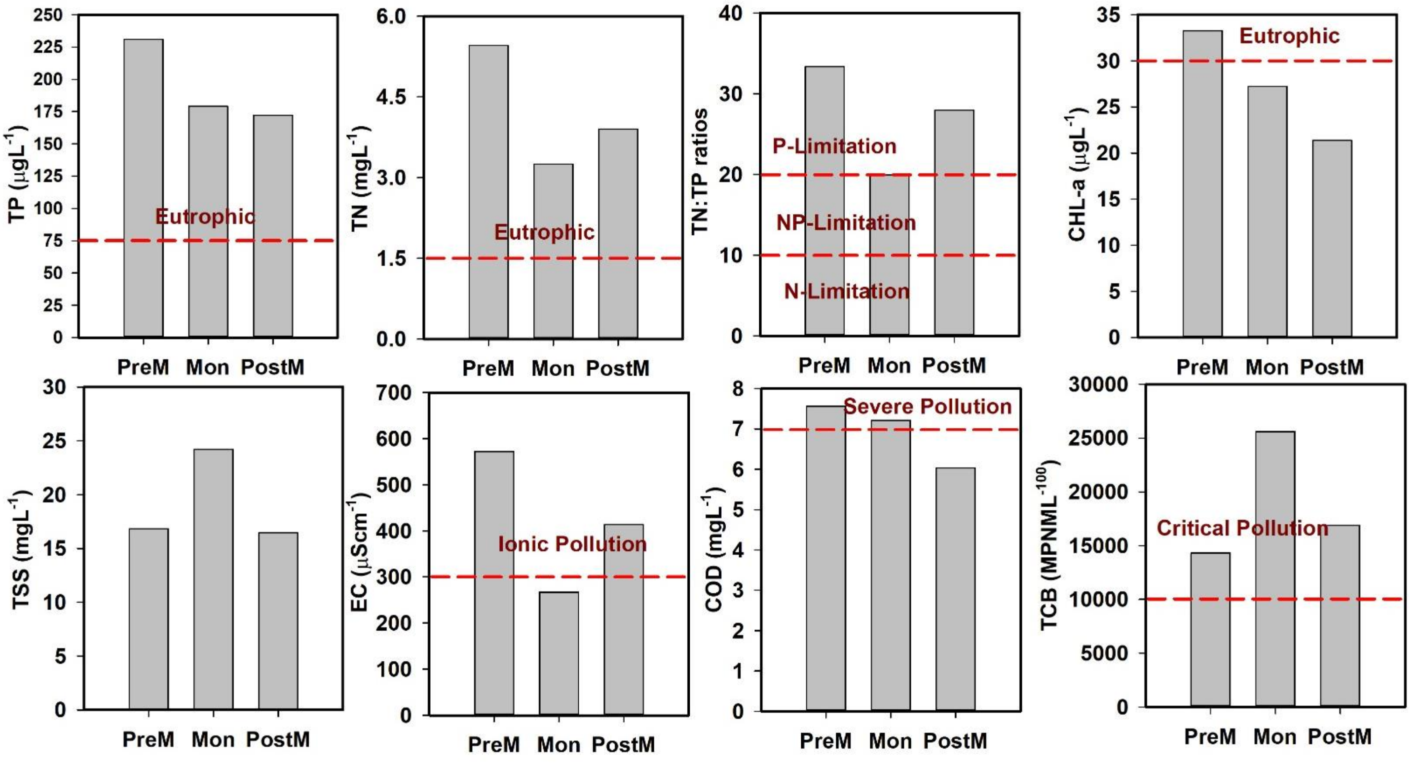

Figure S1. Seasonal impacts on nutrients (TP: total phosphorus, TN: total nitrogen), nutrient ratios (TN:TP ratios), algal chlorophyll (CHL-a: chlorophyll-a), suspended solids (TSS: total suspended solids), ionic concentrations (EC: electrical conductivity), organic matter (COD: chemical oxygen demand) and total coliform bacteria (TCB) in the Yeongsan River (PreM; premonsoon: January-June, Mon; monsoon: July-August, and PostM: postmonsoon: September-December)

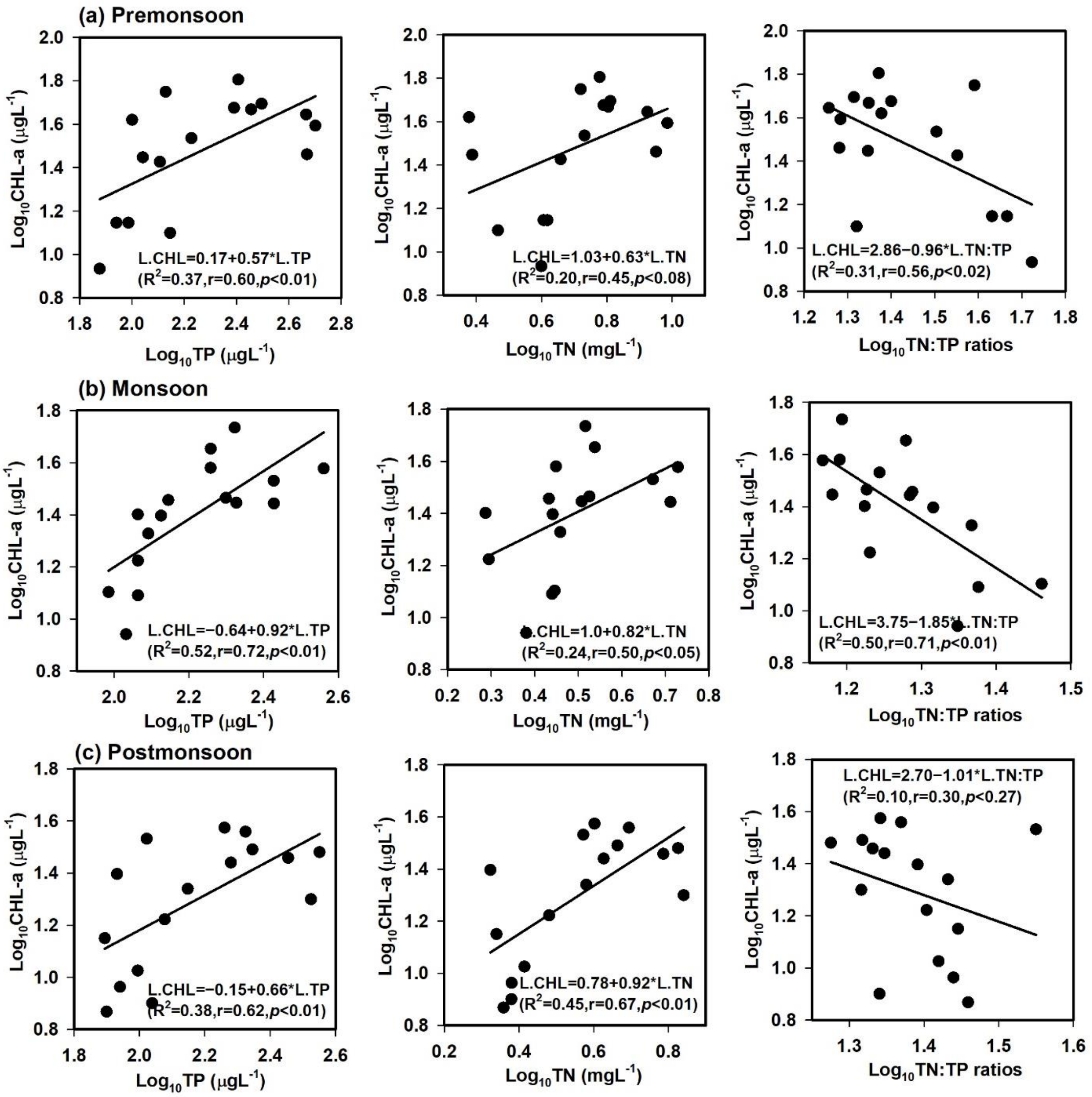

Figure S2. Regression analysis of log-transformed algal chlorphyll (CHL-a: chlorphyll-a) with TP (total phosphorus), TN (total nitrogen), TN:TP ratios during premonsoon (January-June), monsoon (July-August) and postmonsoon (September-December) season in the Yeongsan River

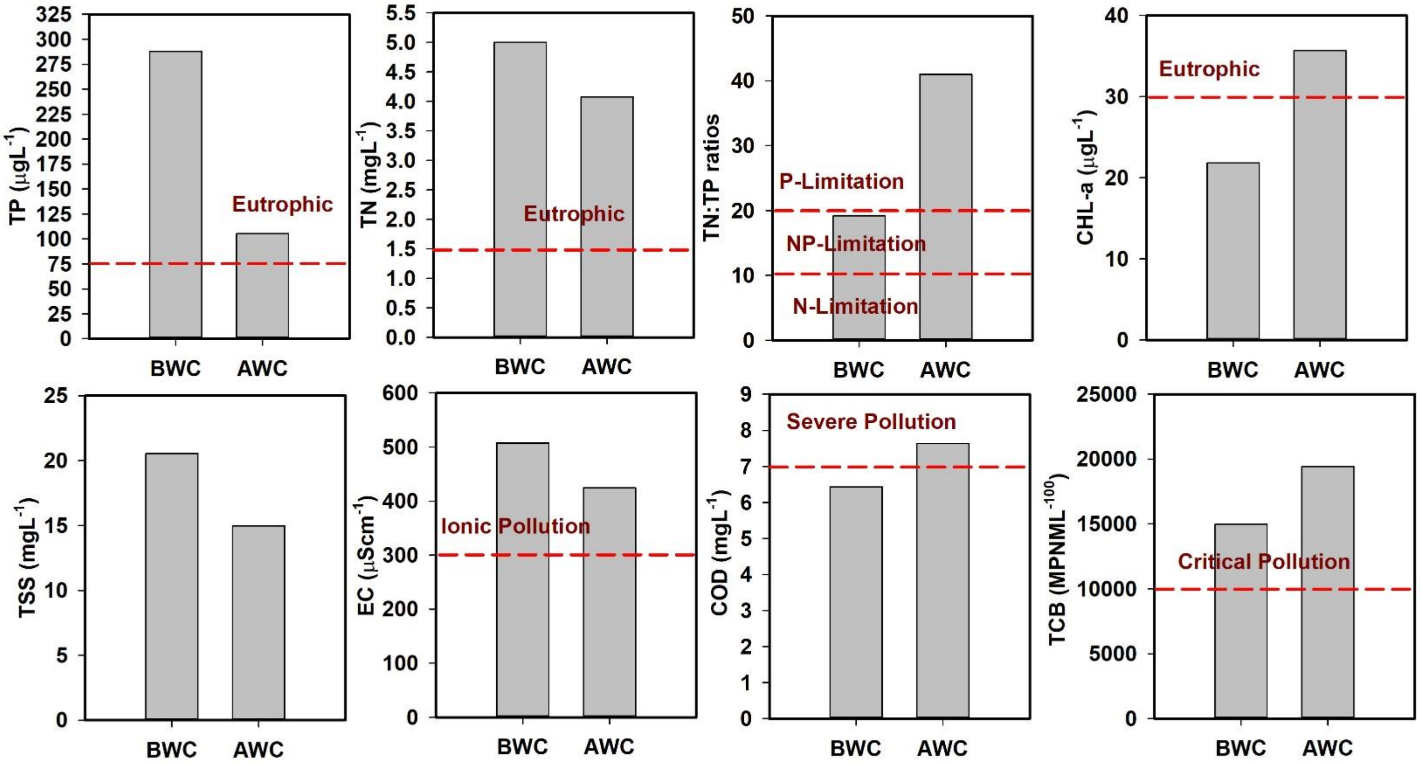

Figure S3. Changes in nutrients (TP: total phosphorus, TN: total nitrogen), nutrient ratios (TN:TP ratios), algal chlorophyll (CHL-a: chlorophyll-a), suspended solids (TSS: total suspended solids), ionic concentrations (EC: electrical conductivity), organic matter (COD: chemical oxygen demand) and total coliform bacteria (TCB) in the Yeongsan River in the periods of before and after weir construction (BWC: before weir construction and AWC: after weir construction)

.

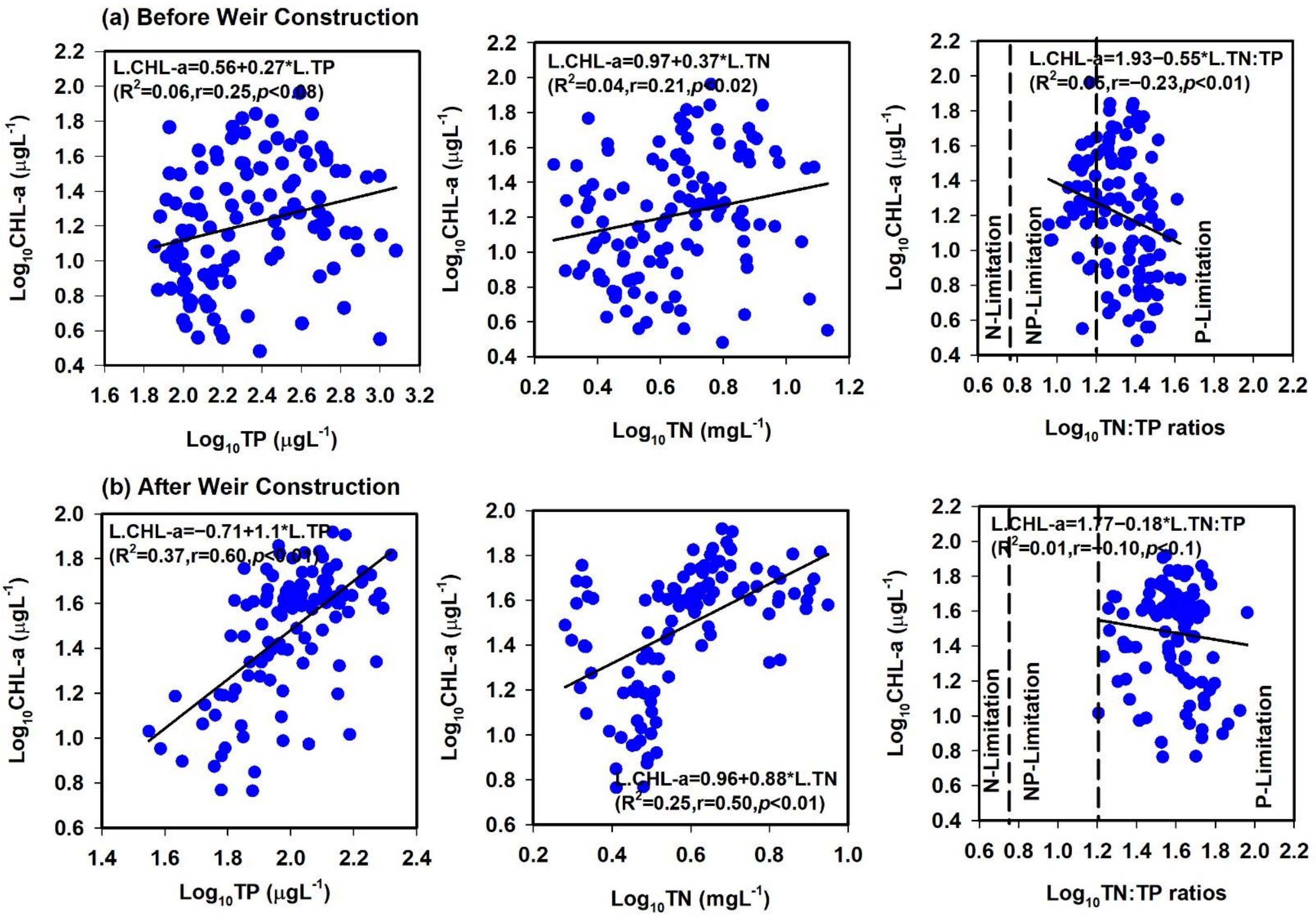

Figure S4. Regression analysis of log-transformed algal chlorphyll (CHL-a: chlorphyll-a) with TP (total phosphorus), TN (total nitrogen), TN:TP ratios in the Yeongsan River in the periods of before and after weir construction

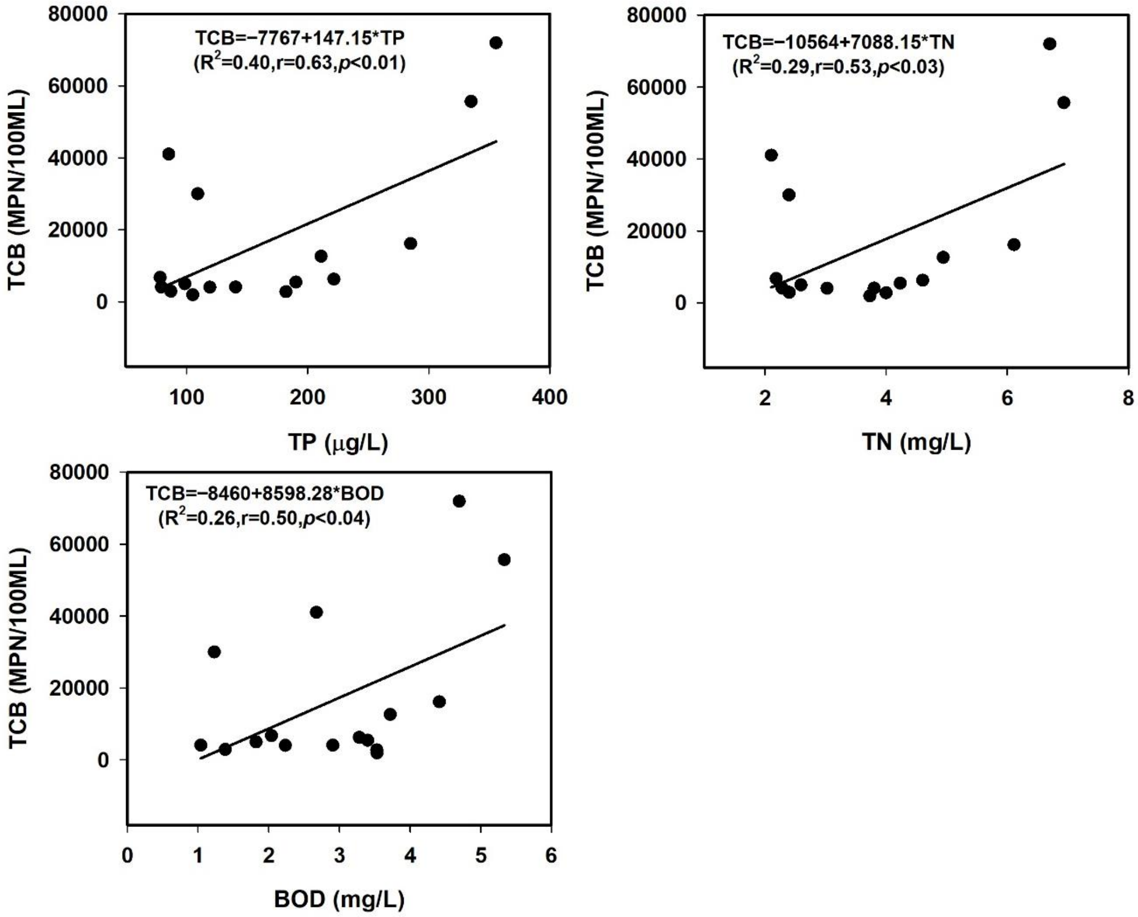

Figure S5. Regression analysis of TCB (total coliform bacteria) with TP (total phosphorus), TN (total nitrogen), and BOD (biological oxygen demand)

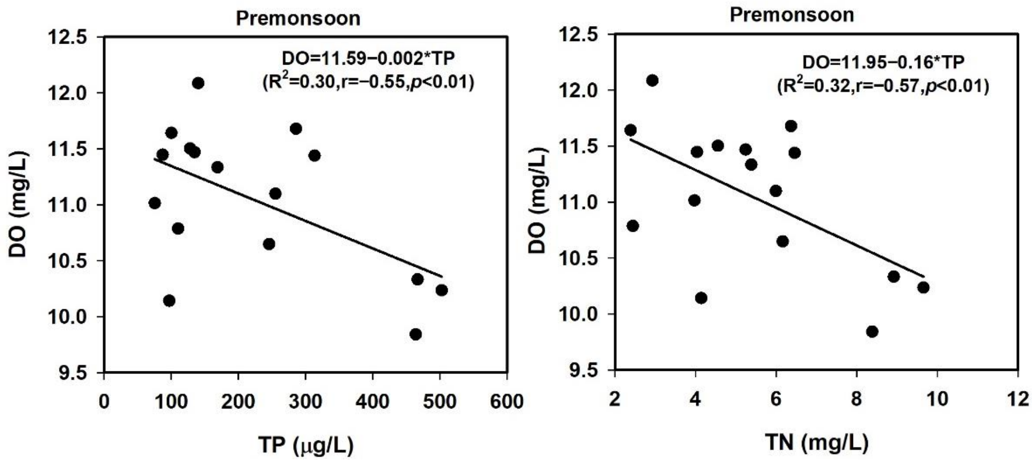

Figure S6. Regression analysis of DO (dissolved oxygen) with TP (total phosphorus) and TN (total nitrogen)

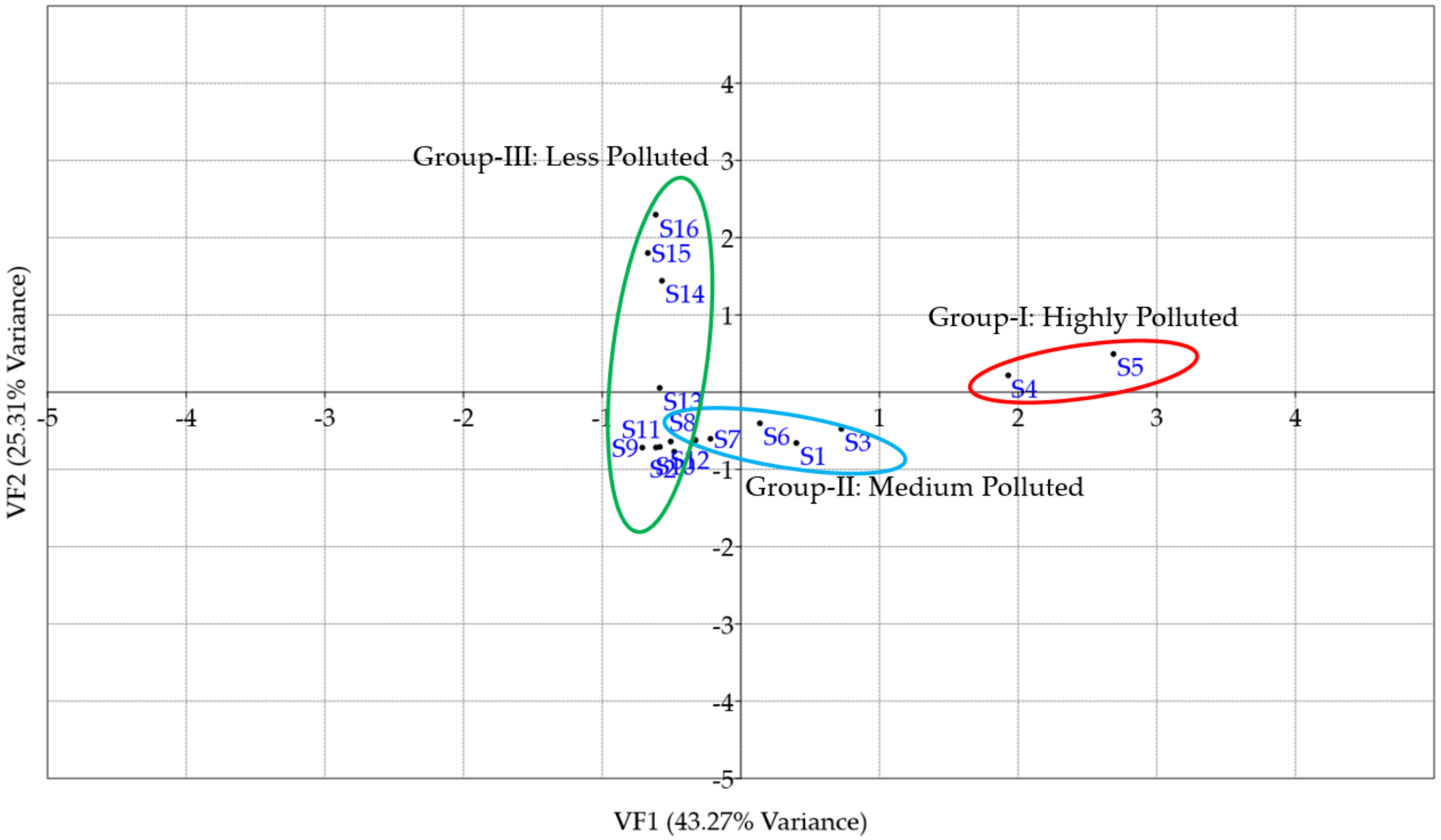

Figure S7. Scatter plot of the eigenvalue scores for the sites of Yeongsan River based on PCA/FA.

Table S1. WQI range, status, and possible usage of the water sample (Brown et al.1972)

Water quality index (WQI)	Water quality status (WQS)	Possible usage
0-25	Excellent	Drinking, irrigation and industrial
26-50	Good	Drinking, irrigation and industrial
51-75	Poor	Irrigation and industrial
76-100	Very poor	Irrigation
Above 100	Unsuitable for drinking and fish culture	Proper treatment required before use

Table S2. The concentration of PO_4-P, TDP, and NO_3-N at 16 sites in Yeongsan River.

Sites	PO_4-P(mg/L)	TDP (mg/L)	NO_3-N(mg/L)
S1	0.08	0.10	1.87
S2	0.04	0.06	1.43
S3	0.03	0.05	1.19
S4	0.25	0.31	3.10
S5	0.27	0.35	3.00
S6	0.22	0.27	3.01
S7	0.16	0.19	2.75
S8	0.14	0.18	2.79
S9	0.05	0.07	2.16
S10	0.12	0.14	2.59
S11	0.11	0.14	2.72
S12	0.08	0.11	2.54
S13	0.06	0.08	2.23
S14	0.05	0.07	2.14
S15	0.04	0.06	2.11
S16	0.04	0.06	2.10

Table S3. Mann-Kendall trend test results for water quality variables at 16 monitoring sites and overall river network (WT: water temperature, EC: electrical conductivity, TP: total phosphorus, TN: total nitrogen, TSS: total suspended solids, BOD: biological oxygen demand, COD: chemical oxygen demand, CHL-a: chlorophyll-a, TCB: total coliform bacteria).

Water Quality Variables	WT	EC	TP	TN	TSS	BOD	COD	CHL-a	TCB
**S1**	↑	↔	↔	↔	↔	↔	↑	↔	↔
**S2**	↔	↑	↔	↓	↔	↔	↔	↔	↔
**S3**	↔	↔	↔	↔	↔	↔	↑	↔	↔
**S4**	↑	↔	↓	↔	↓	↓	↑	↑	↑
**S5**	↑	↔	↓	↔	↔	↔	↑	↑	↑
**S6**	↔	↔	↓	↓	↓	↓	↑	↑	↔
**S7**	↔	↔	↓	↓	↔	↔	↑	↑	↔
**S8**	↔	↔	↓	↓	↔	↔	↑	↑	↓
**S9**	↔	↔	↔	↔	↔	↔	↔	↔	↑
**S10**	↔	↔	↓	↓	↓	↓	↔	↔	↔
**S11**	↔	↔	↓	↓	↓	↔	↑	↔	↔
**S12**	↔	↔	↓	↓	↔	↓	↑	↑	↔
**S13**	↔	↓	↓	↓	↔	↔	↑	↑	↔
**S14**	↔	↔	↓	↓	↔	↔	↑	↑	↓
**S15**	↔	↔	↓	↓	↔	↔	↑	↔	↔
**S16**	↔	↔	↓	↓	↔	↔	↑	↑	↓
**Overall River**	↔	↔	↓	↓	↔	↓	↑	↑	↔

↑: increasing trend, ↓: decreasing trend, ↔: no trend

Table S4. Classification functions for discriminant analysis of spatial variations in water quality of the Yeongsan River. pH- hydrogen ion concentration, WT: water temperature, DO: dissolved oxygen, EC: electrical conductivity, TSS: total suspended solids, TP: total phosphorus, TN: total nitrogen, BOD: biological oxygen demand, COD: chemical oxygen demand, CHL-a: chlorophyll-a, TCB: total coliform bacteria, HP: highly polluted, MP: medium polluted, LP: less polluted) (Fisher's linear discriminant functions).

Variables	Standard mode			Stepwise mode		
	Sites			Sites		
	HP	MP	LP	HP	MP	LP
pH	77.30	82.91	85.37	100.16	104.77	107.11
WT	25.95	25.04	24.64			
DO	6.29	5.89	4.78	6.10	5.74	4.44
EC	0.01	0.008	0.01			
TSS	0.26	0.29	0.32			
TP	0.02	0.03	0.03			
TN	3.25	2.18	2.27	10.45	9.36	9.36
BOD	-6.71	-8.13	-9.13	-8.42	-8.97	-10.16
COD	6.44	5.83	6.54	9.16	8.21	9.04
CHL-a	-0.77	-0.64	-0.65	-0.65	-0.53	-0.55
TCB	-0.0001	-0.0002	-0.0003	-0.0001	-0.0002	-0.0003
(Constant)	-559.95	-565.84	-568.65	-445.18	-458.07	-463.57

Table S5. Classification matrix for discriminant analysis of spatial variations in water quality of the Yeongsan River. HP: highly polluted, MP: medium polluted, LP: less polluted.

Sites	% correct	Sites assigned by DA		
Standard mode		HP	MP	LP
HP	96.4	27	7	0
MP	80.0	1	60	28
LP	77.8	0	8	98
Total	80.8	28	75	126
Stepwise mode				
HP	96.4	27	8	0
MP	77.3	0	58	23
LP	81.7	1	9	103

Total	82.1	28	75	126

Table S6. Classification functions for discriminant analysis of temporal variations in water quality of the Yeongsan River. pH: hydrogen ion concentration, WT: water temperature, DO: dissolved oxygen, EC: electrical conductivity, TSS: total suspended solids, TP: total phosphorus, TN: total nitrogen, BOD: biological oxygen demand, COD: chemical oxygen demand, CHL-a: chlorophyll-a, TCB: total coliform bacteria, Premonsoon: January-June, Monsoon: July-August, Postmonsoon: September-December. (Fisher's linear discriminant functions).

Variables	Standard mode			Stepwise mode		
	Season			Season		
	Premonsoon	Monsoon	Postmonsoon	Premonsoon	Monsoon	Postmonsoon
pH	265.10	257.83	260.35	177.67	170.39	173.56
WT	0.59	1.24	0.61	-1.37	-0.64	-1.28
DO	2.50	3.94	2.12	-6.39	-4.91	-6.52
EC	-0.02	-0.02	-0.02	0.01	0.01	0.008
TSS	-1.61	-1.39	-1.53	-1.538	-1.336	-1.466
TP	0.31	0.32	.31			
TN	5.45	5.20	5.26			
BOD	-21.19	-23.82	-22.16			
COD	28.19	28.48	27.03	8.10	6.94	6.81
CHL-a	-2.49	-2.44	-2.42			
TCB	-0.0001	-0.0001	-0.0001			
(Constant)	-1098.23	-1068.23	-1049.05	-670.70	-638.20	-630.01

Table S7. Classification matrix for discriminant analysis of temporal variations in water quality of the Yeongsan River. Premonsoon: January-June, Monsoon: July-August, Postmonsoon: September-December.

Sites	% correct	Season assigned by DA		
Standard mode		Premonsoon	Monsoon	Postmonsoon
Premonsoon	85.4	82	0	9
Monsoon	93.8	3	30	11
Postmonsoon	68.8	11	2	44
Total	81.3	96	32	64
Stepwise mode				

Premonsoon	84.4	81	0	13
Monsoon	96.9	4	31	11
Postmonsoon	62.5	11	1	40
Total	80.0	96	32	64
